# Dry Tablet Formulation of PLGA Nanoparticles with a Preocular Applicator for Topical Drug Delivery to the Eye

**DOI:** 10.3390/pharmaceutics11120651

**Published:** 2019-12-04

**Authors:** Woo Mi Ryu, Se-Na Kim, Chang Hee Min, Young Bin Choy

**Affiliations:** 1 Interdisciplinary Program in Bioengineering, College of Engineering, Seoul National University, Seoul 08826, Korea; wmryu@snu.ac.kr (W.M.R.); ddf5209@snu.ac.kr (C.H.M.); 2 Institute of Medical & Biological Engineering, Medical Research Center, Seoul National University, Seoul 03080, Korea; ksn777@snu.ac.kr; 3 Department of Biomedical Engineering, Seoul National University College of Medicine, Seoul 03080, Korea

**Keywords:** PLGA, nanoparticles, alginate, ophthalmic drug delivery, dexamethasone

## Abstract

To enhance ocular drug bioavailability, a rapidly dissolving dry tablet containing alginate and drug-loaded poly(lactic-*co*-glycolic acid) (PLGA) nanoparticles was proposed. For hygienic and easy administration of an accurate drug-dose with this tablet, the use of a preocular applicator was suggested. Herein, a dry tablet was prepared by embedding dexamethasone-loaded PLGA nanoparticles in alginate, which was deposited on the tip of the applicator. The nanoparticles were loaded with 85.45 μg/mg drug and exhibited sustained drug release for 10 h. To evaluate in vivo efficacy, dexamethasone concentration in the aqueous humor was measured after topical administration of the dry tablet, with the applicator, to rabbit eyes and was compared to that achieved with Maxidex^®^, a commercially-available dexamethasone eye drops. When applied with the preocular applicator, the dry tablet containing alginate could be fully detached and delivered to the eye surface. In fact, it showed up to 2 h of nanoparticle retention on the preocular surface due to tear viscosity enhancement, causing an estimated 2.6-fold increase in ocular drug bioavailability compared to Maxidex^®^. Therefore, the preocular applicator combined with a dry alginate tablet containing PLGA nanoparticles can be a promising system for aseptically delivering an accurate dose of ophthalmic drug with enhanced bioavailability.

## 1. Introduction

Topical drug administration is an easy route for ocular drug delivery. However, an ophthalmic drug, prepared as a solution and suspension, is known to clear very rapidly from the eye surface (<3 min), leading to a short preocular residence time, ultimately limiting drug bioavailability [1,2]. Less than 5% of a topically-applied drug can reach the interior target tissue of the eye [3,4]. Micro- or nano-particles have thus been suggested as ophthalmic drug carriers to achieve prolonged preocular residence time as well as sustained drug release [5]. Nevertheless, when formulated in the aqueous suspension, an additional fluid added to the eye would accelerate tear clearance, which would also expedite the drainage of the drug-loaded particles [6]. Therefore, an alternative strategy has been suggested and it includes a dry tablet formulation, where the drug-loaded particles are embedded in a tablet medium of a rapidly-dissolving polymer [7]. When applied to the eye surface, the tablet medium would dissolve in the tear fluid to free the drug-loaded particles on the preocular surface. The dissolution of the tablet medium would thus increase tear viscosity to delay tear clearance, which in turn would improve the preocular retention of drug-loaded particles. However, applying a dry tablet to the sensitive eye may not be convenient for patients. In addition, this administration route might not be hygienic, as the tablet would be retrieved with the bare fingers [8,9].

Herein, a combined system of a preocular applicator and a rapidly-dissolving dry tablet containing drug-loaded nanoparticles was proposed. The applicator was designed to contain two compartments (i.e., a handle plus a tip to hold the dry tablet). Thus, for administration, a dry tablet on the tip could approach and touch the eye surface while the handle is held by one’s fingers. The tablet would then be separated from the tip and its medium would be dissolved in the tear fluid to free the drug-loaded nanoparticles. This would provide an easy way to topically administer the dry tablet and importantly, allow tablets to remain aseptic during administration.

To test the system and strategy, a preocular applicator using the biocompatible polydimethylsiloxane (PDMS) was prepared in this study. The shape and dimension of the applicator herein was designed to be similar to that of the commercially-available, one-time use applicator of artificial tear fluid based on patients’ familiarity [10]. The commercially-available applicator is basically a container of liquid; however, a dry tablet formulation was loaded at the tip of the applicator proposed in this work. For the formulation, poly(lactic-*co*-glycolic acid) (PLGA) nanoparticles were prepared to be loaded with dexamethasone, a widely used corticosteroid drug for the treatment of eye inflammation [11,12], to achieve sustained drug release. The nanoparticles were then suspended in a solution containing a mixture of water-soluble polymers, polyvinyl alcohol (PVA), and alginate, which was then freeze-dried on top of the applicator tip to produce a rapidly-dissolving dry tablet. PLGA has been widely used for sustained drug delivery due to its high biocompatibility and degradability to non-toxic by-products [13,14]. PVA has already been approved for clinical use in various ophthalmic applications [15,16]. In the present study, alginate was also employed in the tablet medium as a viscosity enhancer of tear fluids. Alginate is a highly-biocompatible polymer [17] that can form a gel in the presence of the multivalent cation, Ca^2+^, which is abundant in the tear fluid [18,19]. Due to this unique property, when dissolved in the tear fluid, alginate can further increase tear viscosity, thereby synergistically improving the preocular retention of the drug-loaded nanoparticles.

Dexamethasone-loaded PLGA nanoparticles (DX/NP) were prepared by the solid-in-oil-in-water (S/O/W) emulsion method and characterized by scanning electron microscopy (SEM) and dynamic light scattering (DLS), which were employed to assess nanoparticle size and morphology. The cytotoxicity of DX/NP was examined using human corneal epithelial cells (HCECs). To examine the effect of alginate on tear viscosity enhancement, two distinct tablet formulations containing DX/NP were prepared with media containing both PVA and alginate (DX/NP AL_TAB) and PVA alone (i.e., without alginate) (DX/NP TAB), respectively. For in vivo experiments, these tablet formulations were applied to rabbit eyes, and drug concentration in the aqueous humor was assessed and compared to the commercially-available ophthalmic dexamethasone medication, Maxidex^®^, (i.e., an aqueous suspension of dexamethasone itself).

## 2. Materials and Methods

### 2.1. Materials

PLGA (lactic acid:glycolic acid = 50:50; i.v. = 48,000) was purchased from Evonik Industry (Essen, Germany). Dexamethasone and Nile red were purchased from Tokyo Chemical Industry (Tokyo, Japan). PVA (87–89% hydrolyzed), phosphate buffered saline (PBS) tablets, tween 80, trifluoroacetic acid (TFA; >99%), and calcium chloride dihydrate (>99%) were obtained from Merck (St. Louis, MO, USA). Dichloromethane (DCM; >99.5%), *N*,*N*-dimethylformamide (DMF; >99.5%), and acetone (>99.5%) were supplied by DaeJung (Siheung-si, Korea). Acetonitrile (ACN; >99.9%) was purchased from J.T. Bakers (Phillisburg, NJ, USA). PDMS (Sylgard 184) was obtained from Sewang Hitech Silicone (Bucheon-si, Korea). Alcaine^®^ (0.5% proparacaine hydrochloride ophthalmic solution) and Maxidex^®^ (0.1% dexamethasone ophthalmic suspension) were purchased from Alcon-Couvreur (Fort Worth, TX, USA).

### 2.2. Preparation of Drug-Loaded Nanoparticles

DX/NP was prepared via S/O/W emulsification [20]. Briefly, 300 mg PLGA and 100 mg dexamethasone were dissolved in 7 mL DCM. The resulting solution was then added to 8 mL of 1% *w/v* PVA solution, which was emulsified with a sonicator (Sonic Dismembrator Model 500, Fisher Scientific, Illkirch-Graffenstaden, France) at 160 W for 10 min. The emulsion was transferred into 100 mL of 1% *w/v* PVA solution at room temperature and stirred at 400 rpm under vacuum (−10 psi) for 60 min for solvent evaporation. The final suspension was then centrifuged at 200 rpm for 10 min, where the precipitates were eliminated and only the suspension at the top was collected. The collected suspension was then washed with deionized (DI) water three times via centrifugation at 13,000 rpm for 10 min. Thereafter, the suspension was lyophilized for 20 h to obtain dry DX/NP. PLGA nanoparticles loaded with Nile red (NR/NP) instead of dexamethasone were also prepared to evaluate its in vivo preocular retention properties. As such, 5 mg Nile Red and 500 mg PLGA were dissolved in 7 mL DCM for emulsification.

### 2.3. Preparation of Tablet-Loaded Preocular Applicators

**Figure 1:**
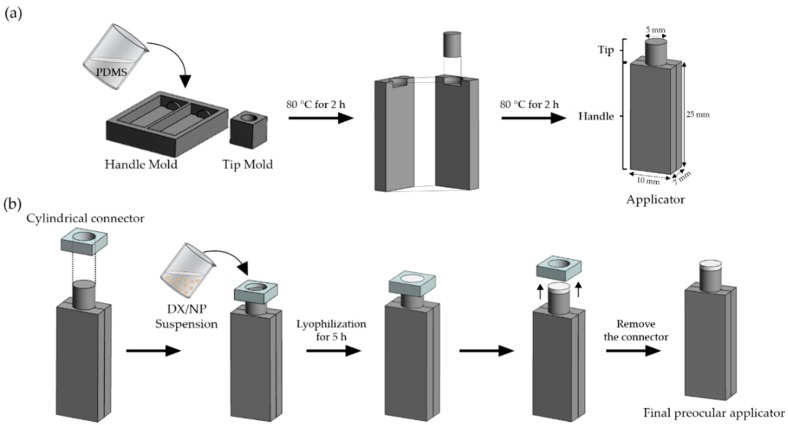
Schematic illustration of the fabrication of the DX/NP tablet-loaded applicator. (**a**) Fabrication of the PDMS applicator. (**b**) Tablet preparation on the tip of the applicator.

A preocular applicator with a dry tablet on the tip was prepared, as depicted in Figure 1. To prepare a preocular applicator, two different molds were first made to fabricate a handle and tip (Figure 1a). In the molds, a mixture of a PDMS base and its curing agent (10:1, *v/v*) was poured and cured slightly at 80 °C for 2 h. Then, each of the constituent pieces was assembled and cured further at 80 °C for another 2 h to allow for their bonding. In this work, two different tablets, with media containing both PVA and alginate, and PVA alone, were produced to yield DX/NP AL_TAB and DX/NP TAB, respectively. To prepare the DX/NP AL_TAB, 20 mg/mL DX/NP were suspended in a solution containing 0.1% *w/v* PVA and 2% *w/v* alginate. Above this alginate concentration, the solution became too viscous to properly distribute the DX/NP. To prepare the DX/NP TAB, 20 mg/mL DX/NP were suspended in a solution of 0.1% *w/v* PVA only. Twenty μL of the resulting suspension was then poured in the reservoir that was made by tightly fitting a cylindrical connector with open ends to a tip of the applicator (Figure 1b). The whole piece was then rapidly frozen using liquid nitrogen, which was then lyophilized for 5 h at 0.01 mbar with the collector temperature set at −80 °C (FreeZone 6 Dryer system, Labconco, Kansas City, MO, USA) [21,22,23]. After that, a connector was carefully removed to produce the preocular applicator loaded with the tablet formulation. To evaluate the in vivo preocular retention properties, NR/NP was embedded instead of DX/NP to produce tablets of NR/NP AL_TAB and NR/NP TAB, prepared under the same condition employed for DX/NP AL_TAB and DX/NP TAB, respectively.

### 2.4. Characterization

The morphology of DX/NP was examined by SEM (JSM-7800F Prime, JEOL, Tokyo, Japan). Its size distribution was determined using DLS (ELS-2000ZS, Otsuka Electronics, Osaka, Japan) and a zetasizer (Nano ZS, Malvern, UK) with a particle suspension prepared in DI water [24]. To measure the drug loading amount, the solution of dexamethasone that was fully extracted from the DX/NP and tablet was respectively prepared. Briefly, 4 mg of DX/NP was completely dissolved in 4 mL DMF. The tablet at the applicator tip was fully immersed in 1 mL DMF, sonicated for 2 h, and centrifuged at 13,000 rpm. The supernatant was then diluted with ACN in a 1:1 ratio. Drug concentration in each resulting solution was measured using high-performance liquid chromatography (HPLC; Agilent 1260 series, Agilent Technologies, Santa Clara, CA, USA) with a Diamonsil C_18_ column (5 μm, 150 × 4.6 mm). Column temperature and absorbance wavelength were set at 37 °C and 240 nm, respectively. Injection volume and flow rate were 20 μL and 1.5 mL/min, respectively. The mobile phase was comprised of 0.1% TFA and ACN mixed in a 65:35 ratio. Given with the loading amount of the DX/NP, the encapsulation efficiency (EE) was calculated by the equation.

(1) $$\mathrm{EE}\;\left(\%\right)=\frac{\mathrm{Amount}\;\mathrm{of}\;\mathrm{drug}\;\mathrm{loaded}\;\mathrm{in}\;\mathrm{nanoparticles}}{\mathrm{Initial}\;\mathrm{amount}\;\mathrm{of}\;\mathrm{drug}}\times 100$$

The thicknesses of the tablets herein were measured using a caliper (ABSOLUTE Digimatic Caliper, Mitutoyo, Kanagawa, Japan). To assess the particle distribution in the tablet, the NR/NP TAB and NR/NP AL_TAB were imaged with a fluorescence microscope (Leica DMI4000 B, Leica Microsystems, Wetzlar, Germany). To examine the effect of the tablet medium, DX/NP AL_TAB and DX/NP TAB were each immersed in 1 mL of the medium mimicking tear fluid, i.e., PBS containing Ca^2+^ (10 mM, pH 7.4, [Ca^2+^] = 39.4 µg/mL) [25], for 10 min at 37 °C. The viscosity of the resulting solution was then measured using a rheometer (Advanced Rheometric Expansion System, Rheometric Scientific, New Castle, DE, USA), where gap separation, temperature, and shear rate were set at 0.8 mm, 25 °C, and 100 s^−1^, respectively.

### 2.5. In Vitro Drug Release Study

To examine the in vitro drug release profiles, 400 μg DX/NP, two different tablets containing the DX/NP (i.e., DX/NP TAB and DX/NP AL_TAB) and Maxidex^®^, all of which contained the same amount of about 35 μg DX were each placed in a dialysis membrane bag (SnakeSkin^TM^ Dialysis Tubing, 10 kDa, Thermo Scientific, USA). The bag was then immersed in 5 mL pH 7.4 PBS containing 39.4 µg/mL Ca^2+^ and 0.5% *w/v* Tween 80 to meet the sink condition of DX [26]. While being incubated at 37 °C, at scheduled times, 1 mL of the release medium was collected and the same volume of fresh PBS was added back. The amount of released dexamethasone was measured using HPLC as described above.

### 2.6. Cytotoxicity Evaluation

The in vitro cytotoxicity of DX/NP was evaluated using HCECs (PCS-700-010, ATCC, Manassas, VA, USA). HCECs were grown in a corneal epithelial cell basal medium (PCS-700-030, ATCC, USA) with supplements (PCS-700-040, ATCC) at 37 °C in a humidified environment with 5% CO_2_. Prior to the assay, HCECs were seeded in a 96-well plate at 1 × 10^5^ cells/well and grown for 24 h. Subsequently, 100 μL of the DX/NP suspension, which was prepared in the cell growth medium at concentrations of 5, 10, 25, 50, 100, 250, 500, and 1000 μg/mL, was added to each well and incubated at 37 °C for 24 h. The medium was then completely removed and replaced with 100 μL of fresh medium. Thereafter, 10 μL of an EZ-Cytox solution was added to each well and incubated at 37 °C for 2 h under dark conditions. Cell viability was measured using a microplate reader, with absorbance and reference wavelengths of 450 nm and 600 nm, respectively (VersaMax ELISA Microplate Reader; Molecular Devices, San Jose, CA, USA).

### 2.7. Animal Experiments

In vivo experiments were conducted with the healthy eyes of male New Zealand White rabbits (weight 2.1–2.5 kg). Rabbits were granted free access to food and water and were housed in a controlled environment: temperature; 21 ± 1 °C, humidity; 55 ± 1%, and light/dark cycle; 12 h/12 h. The in vivo experimental protocols were approved by the Institutional Animal Care and Use Committee at Seoul National University Hospital (IACUC No. 19–0133, date: 30 July 2019).

First, the in vivo preocular retention properties of the nanoparticles were evaluated after topical administration to the eye. The preocular retention properties were assessed as reported in our previous studies, with slight modifications [27,28]. Briefly, the NR/NP AL_TAB or NR/NP TAB on the applicator tip was applied on the lower cul-de-sac of the left eye of rabbits. At scheduled times, the rabbit eye was anesthetized by topical administration of a drop of Alcaine^®^ and the entire preocular surface was thoroughly wiped with a surgical sponge (PVA spear; Sidapharm, Thessaloniki, Greece) to collect the NR/NP. The sponge was then fully immersed in 5 mL DMF and sonicated for 2 h to fully dissolve the NR/NP. The amount of Nile red in the sample was measured using HPLC-mass spectroscopy (LC-MS) with a Polaris 5 C_18_-A (2.7 µm pore size, 4.6 × 150 mm) and the following conditions: column temperature, 30 °C; absorbance wavelength, 243 nm; injection volume, 20 μL; and flow rate, 0.45 mL/min. The mobile phase was prepared by mixing 0.1% formic acid and ACN in the ratio, 55:45. For statistics, four animals (i.e., one left eye for each rabbit) were assigned per time point for each formulation. With the NR/NP AL_TAB and NR/NP TAB, the in vivo profile of tablet disintegration was also assessed. For this, the eye was imaged using a digital camera (Galaxy S10, Samsung, Seoul, Korea) at 0 and 30 s after topical administration of the tablet to rabbit eyes.

To assess the in vivo ocular drug bioavailability, each of the three formulations (i.e., the DX/NP AL_TAB and DX/NP TAB in the applicator, and 35 μL Maxidex^®^) with the same dose of dexamethasone (ca. 35 μg dexamethasone) was administered onto the lower cul-de-sac of rabbit eyes. At scheduled times, the rabbit was anesthetized with a subcutaneous injection of a cocktail containing 20 mg/kg ketamine and 10 mg/kg xylazine. Thereafter, approximately 100 μL of aqueous humor (AH) was collected using a 31 G needle (BD Ultra-Fine II, Becton Dickinson and Company, Franklin Lakes, NJ, USA). Drug concentration in AH was analyzed by LC-MS as described above. For statistics, three animals (i.e., the left eye of each rabbit) were assigned per time point for each formulation.

### 2.8. Statistical Analysis

Statistical analysis was performed using the amount of particles remaining on the preocular surface and drug concentration in AH by the Mann–Whitney U-test. A *p*-value < 0.05 was considered to indicate statistical significance (SPSS version 22, IBM, Armonk, NY, USA).

## 3. Results

### 3.1. Characterization of the Formulations

**Figure 2:**
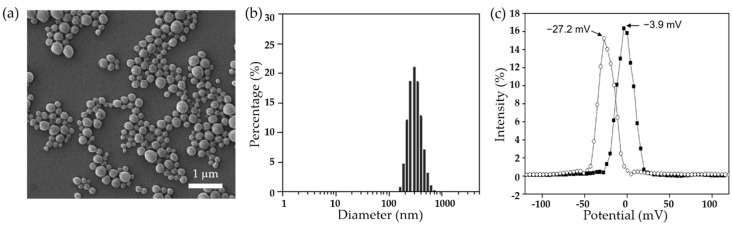
Characterization of DX/NP. (**a**) Representative scanning electron micrographs of DX/NP. (**b**) Particle size distribution of DX/NP measured by the dynamic light scattering (DLS) method; Polydispersity index = 0.056. (**c**) Zeta potentials of the blank PLGA nanoparticles (■) and DX/NP (○).

**Figure 3:**
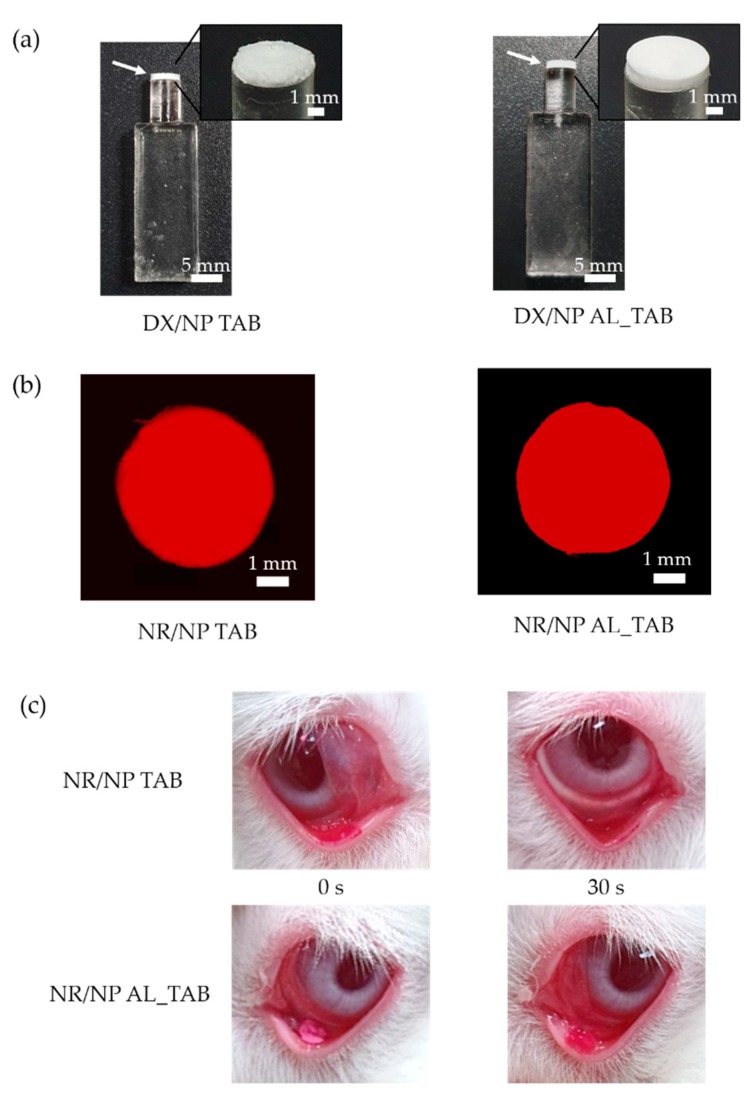
Characterizations of tablets. (**a**) Representative optical images of the applicator with the DX/NP TAB and DX/NP AL_TAB. White arrows indicate the dry tablets prepared on the applicator tip. (**b**) Representative fluorescence images of the NR/NP TAB and NR/NP AL_TAB. (**c**) Disintegration profiles of the NR/NP TAB and NR/NP AL_TAB administered topically to rabbit eyes.

DX/NP was prepared using the emulsion method. As a result, the drug-loaded nanoparticles were found to display a spherical shape, as shown in Figure 2a. Particle diameter was 336.92 ± 5.56 nm (Figure 2b) while drug-loading amount was 85.45 ± 5.44 μg/mg (i.e., EE = c.a. 25.6%). The size and shape of NR/NP were similar to those of DX/NP (Figure S1 in the Supplementary Information). As shown in Figure 2c, the zeta potential of the blank PLGA nanoparticles was measured to be −3.9 mV, which was shifted to −27.2 mV with the DX/NP due to a negative charge of the encapsulated drug, DX [29]. Figure 3a shows the tablets prepared in the present experiment (i.e., the DX/NP TAB and DX/NP AL_TAB). They displayed a cylindrical shape and were well attached at the tip applicator. As shown in Figure 3b, the tablets loaded with the NR/NP exhibited a evenly-distributed fluorescent signal throughout the medium, implying a homogenous distribution of the particles in the tablet. As the same amount of DX/NP was embedded during tablet preparation, the drug loading amount per tablet was similar between DX/NP TAB and DX/NP AL_TAB (34.11 ± 0.48 and 34.89 ± 0.28 μg, respectively). For the same reason, the thicknesses of the DX/NP TAB and DX/NP AL_TAB were also similar, which were measured to be 1.02 ± 0.03 and 1.02 ± 0.06 mm, respectively. When tablets were dissolved in Ca^2+^-containing PBS, the solution with DX/NP AL_TAB (i.e., the tablet containing 400 μg alginate) had a higher viscosity of 0.93 Pa s than that with DX/NP TAB (i.e., the tablet without alginate) of 0.01 Pa s. However, both tablets herein were observed to be disintegrated in 30 s by dissolution in tear fluid when topically administered to rabbit eyes (Figure 3c).

### 3.2. In Vitro Evaluation Results

**Figure 4:**
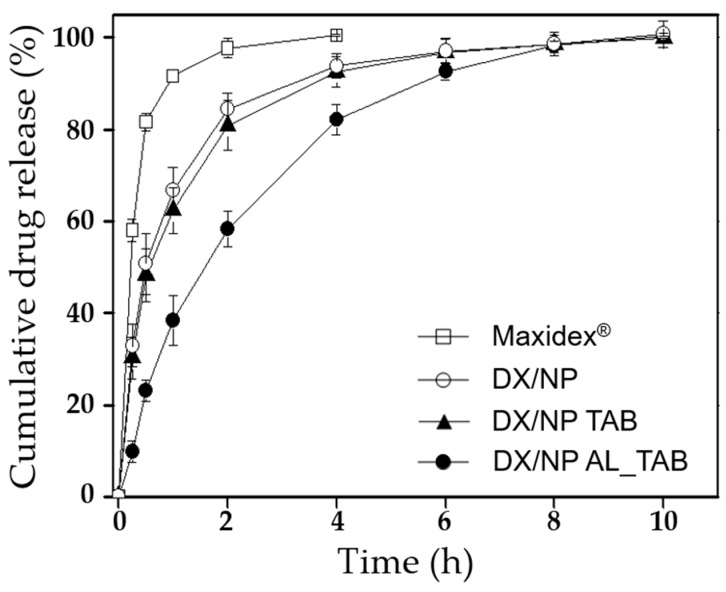
In vitro drug release profiles of Maxidex^®^ (□), DX/NP (○), DX/NP TAB (▲), and DX/NP AL_TAB (●).

**Figure 5:**
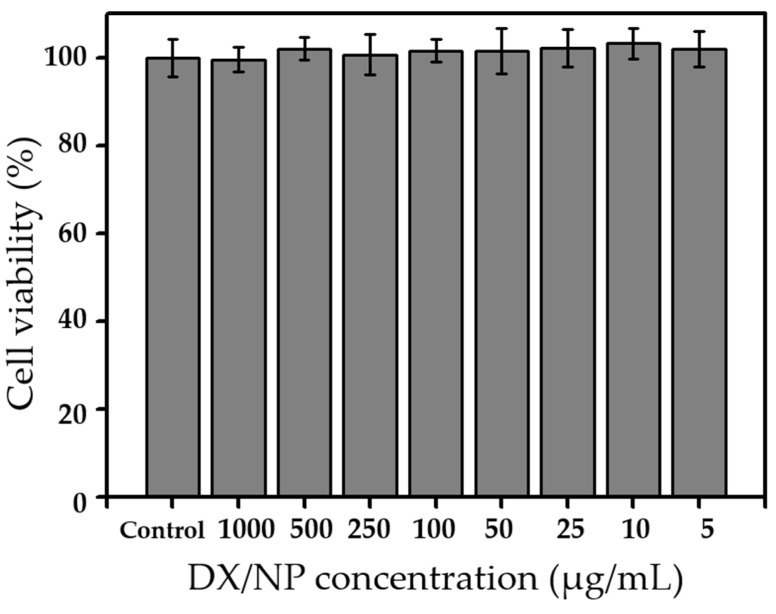
In vitro cytotoxicity of DX/NP on human primary corneal epithelial cells.

Figure 4 shows the in vitro drug release profiles of DX/NP and the tablet formulations, which displayed sustained drug release for up to 10 h. The release profiles did not differ among the formulations as the tablet medium dissolved rapidly to achieve almost instantaneous freeing of DX/NP. Drug release was slightly more suppressed with DX/NP AL_TAB, which could be due to a slight increase in the viscosity of the release medium in a dialysis membrane bag owing to the interaction between algnate and Ca^2+^. On the other hand, Maxidex^®^, which is basically a suspension of DX, exhibited almost complete dissolution of the drug in 2 h. When tested with HCECs, the DX/NP exhibited a cell viability greater than 90% at all testing concentrations (Figure 5), suggesting that the DX/NP was non-cytotoxic.

### 3.3. In Vivo Experimental Results

**Figure 6:**
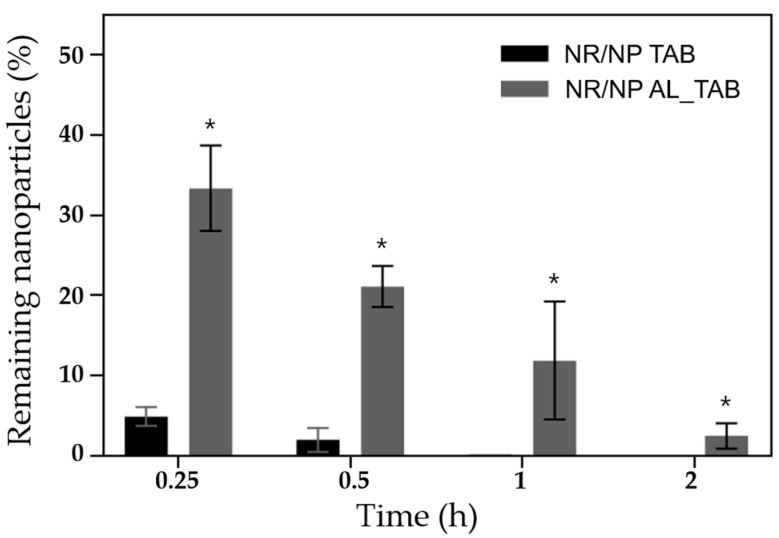
In vivo preocular retention profiles of NR/NP TAB (■) and NR/NP AL_TAB (■) on rabbit eyes. * indicated statistical significance between the formulations at each time point (*p* < 0.05).

To examine the effect of alginate on preocular retention of the nanoparticles, two different tablets embedded with the Nile red-loaded nanoparticles (i.e., NR/NP TAB and NR/NP AL_TAB) were employed for in vivo evaluation. As shown in Figure 6, by incorporating alginate in the tablet, the amount of nanoparticles remaining at the preocular surface increased. For the tablet without alginate, the average percent of remaining particles was relatively low (i.e., 4.8 and 0.2% at 15 min and 1 h, respectively). However, owing to the presence of alginate in the tablet, 33 and 12% of particles remained at 15 min and 1 h, respectively, and could still be detected until 2 h. As conventional eye drops are known to completely disappear in 5 min [1], such findings suggest a greater improvement in drug retention at the eye surface. For the tablet with alginate, there was a high variability in particle retention observed at 1 h. Right after the tablet medium dissolved in the tear fluid to interact with Ca^2+^ ions, the tear viscosity increased to allow for an evident retention of the NP at the preocular surface. However, as the time progressed, the tear fluid with alginate would be continuously diluted with a newly-generated fresh tear, which would vary greatly depending on the subject. After 2 h, almost all particles would be cleared from the preocular surface.

**Figure 7:**
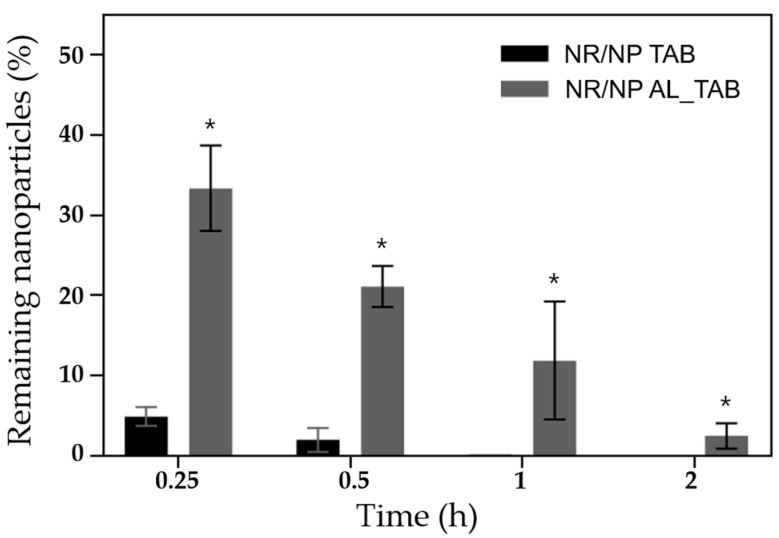
Dexamethasone concentration in the AH of rabbit eyes after the administration of Maxidex^®^ (○), DX/NP TAB (□), and DX/NP AL_TAB (▲). * DX/NP AL_TAB was significantly different from Maxidex^®^.

The pharmacokinetic profiles of the tablet formulations were assessed and compared with that of the commercially-available dexamethasone eye drops, Maxidex^®^. Figure 7 and Table 1 display the drug concentration profiles in the AH and their pharmacokinetic parameters, respectively. At 30 min, Maxidex^®^ had a C_max_ of 285.93 ng/mL; however, this value rapidly decreased to an undetectable level at 6 h after the administration. Interestingly, albeit formulated in a dry tablet, the DX/NP TAB without alginate exhibited a much lower drug bioavailability. In addition, its area under the drug concentration-time curve (AUC) and Cmax were less than a half of those with Maxidex^®^. Such finding could be due to a lower amount of drug exposure at the eye surface, where the DX/NP disappeared relatively rapidly and only a small portion of drug within the particles was actually released during the early period post-administration. On the other hand, 100% drug would be exposed right after a bolus administration with Maxidex^®^.

The highest drug bioavailability was observed with the DX/NP AL_TAB. In fact, its AUC was 2.5-fold greater than that of Maxidex^®^. By incorporating alginate in the tablet, it appeared to increase the viscosity of the tear fluid, enabling a larger amount of DX/NP to be retained in the preocular space for a longer period. During this period, the DX/NP would release and expose a significant amount of dexamethasone in a sustained manner. As a result, the drug concentration in tear would gradually increase before being absorbed into the AH to shift the time to reach C_max_ (T_max_) to a later point (i.e., 2 h) and further increase the value of C_max_ itself, compared to that of Maxidex^®^. Due to prolonged retention and sustained drug release, drug clearance from the AH was delayed, demonstrating that drug concentration in the AH was statistically significantly higher than that of Maxidex^®^ at 4–10 h (*p* < 0.05). All tablets tested in this experiment were almost instantaneously fully detached and released from the applicator tip to the preocular surface when they were in contact with the lower cul-de-sac of rabbit eyes (Figure S2 in Supplementary Information).

## 4. Discussion

Topical drug administration is considered to be an easy route for ophthalmic drug delivery. However, for conventional formulations of drops and suspensions, the additional liquid accelerates tear clearance to further lower drug bioavailability in the eye [30,31]. To overcome this, a dry tablet loaded with microparticles had been proposed as topical drug-delivery formulations [7]. The tablet was prepared by compressing the medium of mannitol together with drug-loaded microparticles composed of PLGA and a mucoadhesion promoter, PEG. Thus, dissolution of mannitol increased the tear viscosity to allow for a prolonged time of interaction between PEG in particles and mucin in the preocular surface. However, the tablet with a high density appeared to dissolve in tear fluid in minutes, during which a relatively large tablet could cause eye irritation. Therefore, the formulation was upgraded to be able to dissolve rapidly in tear fluid and for this, the dry tablet was prepared by freeze-drying [7]. In this way, the tablet medium could possess a porous structure and thus, after administration, it dissolved almost instantaneously in tear fluid to free the drug-loaded particles in the preocular surface. With this strategy, the particles still maintained a preocular retention property, as well as showing sustained drug release, hence improved drug bioavailability in the eye.

Herein, a rapidly-dissolving dry tablet formulation embedded with drug-loaded PLGA nanoparticles (i.e., the DX/NP) was proposed again for topical drug delivery to the eye. Owing to the presence of alginate in the tablet medium, tear viscosity increased because of the interaction between alginate and Ca^2+^. This hampered tear clearance, leading to a higher preocular retention of DX/NP (Figure 6). Therefore, in addition to residing longer in the preocular space, DX/NP could continuously release the drug to tear fluids, providing more time for drug diffusion into the eye and eventually improving ocular drug bioavailability (Figure 7).

In the present study, the tablets were prepared by freeze-drying the DX/NP suspension; this allowed the tablet medium of the water-soluble polymers to be highly porous for rapid dissolution of the tablet in the tear fluid. Considering commercialization, the moisture content in the tablet may need to be minimized further to provide with an appropriate shelf-life of the product. Owing to the nano-sizes of DX/NP, the sensitive eye surface would not be irritated [32]. Therefore, with the alginate content employed for the tablet herein, any sign of eye irritation or discomfort was observed during in vivo experiments. By using the applicator, the tablet could be delivered without being touched, thereby achieving a hygienic application. PDMS was employed as the constituent material for the applicator because of its biocompatibility, should the applicator touch the eye surface. More importantly, PDMS is inherently hydrophobic and it is known to possess a low surface release energy [33]. Therefore, the dry tablet composed of hydrophilic polymers could be separated almost instantaneously without loss when wet with tear fluids (Figure S2), thereby enabling the delivery of an accurate drug dose to the eye using the tablet formulation.

## 5. Conclusions

Herein, a formulation consisting of a dry tablet containing alginate and drug-loaded PLGA nanoparticles is derived to improve the bioavailability of drugs that are topically delivered to the eye. Owing to the ease and hygienic administration of the developed tablet formulation, the use of a preocular applicator is suggested. Based on the hydrophobicity and low surface release energy of the constituent material, PDMS, the applicator could almost instantaneously cause full separation of the dry tablet composed of hydrophilic polymers when applied to the eye surface. After topical delivery, the tablet medium dissolved rapidly to free the drug-loaded nanoparticles in the tear fluid, where alginate could react with Ca^2+^ to increase its viscosity. Such findings suggest that the drug-loaded PLGA nanoparticles can be better retained in the preocular space and the drug can be subsequently released in a sustained manner, ultimately enhancing ocular drug bioavailability. Therefore, it is concluded that the combination of a dry tablet formulation with an alginate medium, drug-loaded PLGA nanoparticles, and a preocular applicator is a promising strategy to achieve patient-friendly, topical drug delivery to the eye, with enhanced drug availability.

## Figures and Tables

**Table 1:** Pharmacokinetic parameters of dexamethasone in the AH.

Formulations	T_max_ (h)	C_max_ (ng·mL^−1^)	AUC ^a^ (ng·h·mL^−1^)
Maxidex^®^	0.5	285.93	388.51
DX/NP TAB	1	62.18	165.93
DX/NP AL_TAB	2	370.33	981.23

**^a^** calculated using the trapezoidal rule.

## Supplementary Materials

The following are available online at https://www.mdpi.com/1999-4923/11/12/651/s1; Figure S1: Characterizations of NR/NP; Figure S2: Images of rabbit eyes.
