# Supplementary material for: Dry Tablet Formulation of PLGA Nanoparticles with a Preocular Applicator for Topical Drug Delivery to the Eye
Source: Pharmaceutics. 2019 Dec 4;11(12):651. doi: 10.3390/pharmaceutics11120651 (PMC6955998; doi:10.3390/pharmaceutics11120651)
Supplement: Supplementary file 1 [file pharmaceutics-11-00651-s001.pdf]

pharmaceutics

Supplementary Materials: Dry Tablet Formulation of PLGA Nanoparticles with a Preocular Applicator for Topical Drug Delivery to the Eye

Woo Mi Ryu, Se-Na Kim, Chang Hee Min, and Young Bin Choy

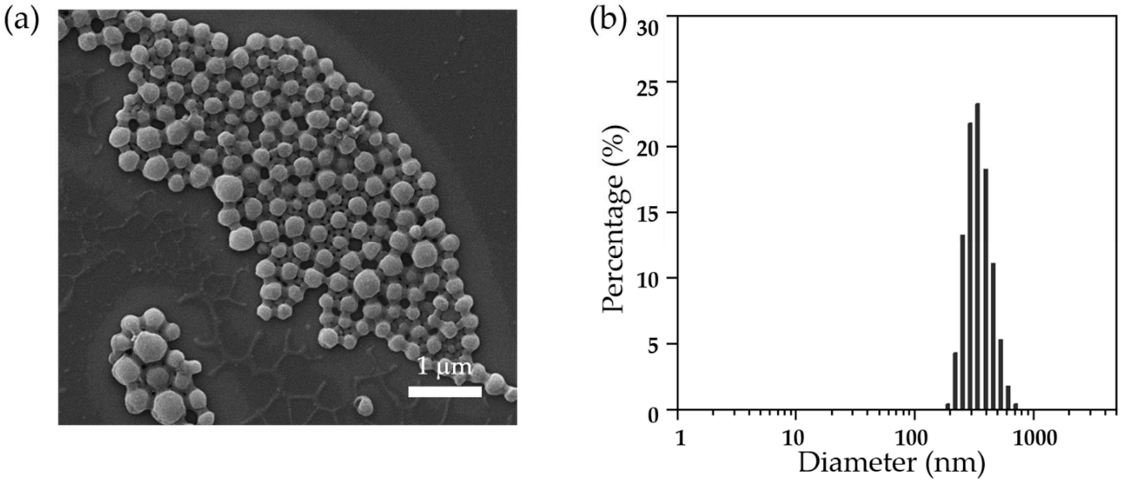

Figure S1. Characterization of NR/NP. (**a**) Representative scanning electron micrographs. (**b**) Dynamic light scattering result for size distribution. Polydispersity index = 0.158. The sizes of the NR/NP were similar to those of DX/NP.

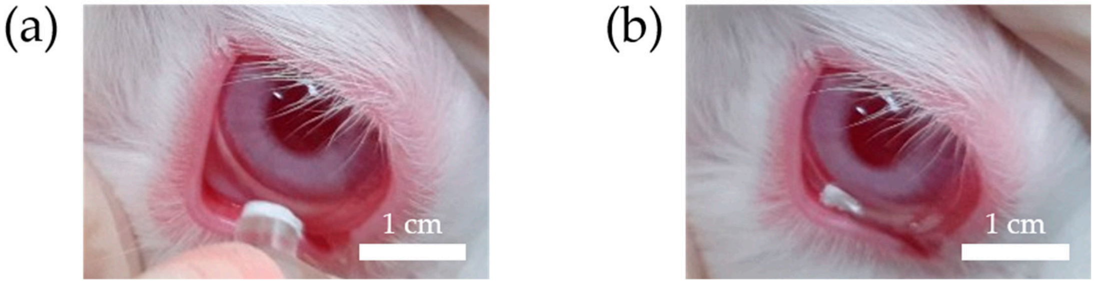

Figure S2. Images of rabbit eyes (**a**) before and (**b**) right after topical administration of the dry tablet using the applicator herein.
